# Impact of cell fusion in myeloma marrow microenvironment on tumor progression

**DOI:** 10.18632/oncotarget.25742

**Published:** 2018-07-24

**Authors:** Ziyan Wang, Yuqing Yuan, Liying Zhang, Zhou Min, Dongming Zhou, Sun Yu, Panjun Wang, Songguang Ju, Li Jun, Jinxiang Fu

**Affiliations:** ^1^ Hematology Department, The Second Affiliated Hospital of Soochow University, Suzhou 215004, PR China; ^2^ Institute of Biotechnology, Soochow University, Suzhou 215007, PR China

**Keywords:** multiple myeloma, bone marrow microenvironment, mesenchymal stem cell, cell fusion, chemoresistance

## Abstract

**Background:**

Mesenchymal stem cells (MSCs) represent a subset of non-hematopoietic adult stem cells, which can also fuse with other cells spontaneously in bone marrow and capable of adopting the phenotype of other cells. The fusion of somatic cells with stem cells can reprogram somatic cells to a pluripotent state. Our research on the fusion of bone marrow mesenchymal stem cells(BM-MSCs) and MM cells demonstrate that the fused cells can exhibit stemness and cancer cell-like characteristics.

**Results:**

We successfully produced a hybrid cells that acquired larger size and multinucleation, in which partial chromatin condensation, a visible nucleolus, and one or more round or oval nucleus. Experiments results showed that the stemness markers highly expressed in these fused cells and there were much more chromosomes in fused cells than those in parental cells as well as exhibited increased resistance to drug treatment.

**Conclusions:**

Our results suggest that cell fusion between BM-MSCs and MM cells could contribute it genomic heterogeneity and play a role on disease progression.

**Methods:**

We fused human BM-MSCs with MM cells lines RPMI 8226 or XG1 *in vitro* by polyethylene glycol (PEG), and the hybrid cells were sorted by sedimentation assays. The growth, migration, cell cycle, chromosome and drug sensitive of hybrids were assessed by cell counting, cell colony formation, transwell assays, cytogenetic assay and flow cytometry (FCM). The proteins and genes related to stemness and cytokines were tested by western blot and/or real-time quantitative RT-PCR.

## INTRODUCTION

Multiple myeloma (MM) is a lethal B cell neoplasm characterized by the monoclonal expansion of malignant plasma cells in the bone marrow microenvironment, resulting in gross skeletal devastation, hypercalcemia, renal failure, and end-organ sequelae [1, 2]. Some abnormalities start at the time of initial transformation, while some occur later in the disease course as the malignancy progresses to a more relapsed refractory state [3–5]. Although several lines of evidence support the existence of a variety of chromosomal aberrations, translocations, and mutations in essential growth and tumor suppressor genes in MM cells, there is more than 50% of patients exhibit a normal karyotype [6–7], thus revealing its marked genomic heterogeneity. There are two broad types of cytogenetic abnormalities in MM: primary and secondary. Primary cytogenetic abnormalities are thought to occur at the time of monoclonal gammopathy of undetermined significance (MGUS) and are believed to have a great impact on pathogenesis of MGUS/MM. But the secondary cytogenetic abnormalities are overlapping and change in MM progression, together with the heterogeneity of both condition, make it difficult to precise definition and the influence disease outcome [8, 9].

MM cells home from lymph nodes to bone marrow through the CXCR4/SDF1 axis [10, 11] and adhere to stromal cells via multiple cell-surface molecules. Studies have demonstrated that extensive connections between MM cells, cellular and extracellular bone marrow elements promote tumor growth, survival, migration, and drug resistance, thus significantly contributing to disease progression [12, 13]. In particular, it has been inferred that the progressive gain of genetic and epigenetic modifications in both myeloma and bone marrow-resident cells is strictly required for driving MM [14–16].

Cell fusion, as a process in which two or more cells become one and generate a new cellular element, is a strictly regulated and plays critical roles in several physiological and pathophysiological events including fertilization, tissue regeneration and viral infection. In the earlier 1990s, the cell fusion has been postulated to play a role in cancerogenesis and proposed that this process could give rise to new cells with chromosomal abnormalities and tumorigenic potential. The presence of large atypical tumor cells with multiple copies of DNAs is refereed to giant cancer cells, which may display a certain level of heterogeneity. Recent data domentrated [17, 18] that the cell fusion per se may account for both the genotypic and phenotypic diversities of different tumor types. The hybridization of tumor cells with normal cells could result in malignant cellular elements with higher metastatic potential. At a molecular level, however, the new multinucleated cells formed by the fusion of single cell including different steps such as cell–cell recognition, adhesion, and membrane integration remain a poorly understood process and are still considered to be one of the key factors in the tumor microenvironment.

BM-MSCs are one of an essential component of bone marrow niche. It has been inferred that a crosstalk/fusion between MM cells and BM-MSCs may be involved in the malignant transformation of the bone marrow microenvironment. Recently, it has become clear that the bone marrow niche is required for driving MM and has significant impact on MM biology [19, 20]. Studies have demonstrated that BM-MSCs could merge with other cells in solid tumors, such as pre-malignant cells or cancer cells, and play an important role in the occurrence of tumor. BM-MSCs are considered as a promising fusogenic candidate in MM microenvironment, whether these phenomena occur and play a role in pathogenesis of MM? Recent studies [14–16] have shown that the fusion of pre-malignant cells with stem cells are more malignant than the parental cells and gain self-renewal and migratory abilities, which highlight the pro-tumor role of stem cells by fusing with other cells. Of particular interest to us was the role of cell fusion in the bone marrow. In the present studies we aimed to explore the role of cell fusion between BM-MSCs and MM cells in proliferation, drug resistant and apoptosis *in vitro*. By *in vitro* using an co-culture research model we showed that BM-MSCs and MM cells were fused in medium containing polyethyleneglycol-1000 (PEG-1000). The resulting cells seemed to have more aggressive behavior and the expression of stem cells related to transcription factors Oct4, c-Myc, Sox2 and Nanog was also investigated in these fused cells.

## RESULTS

### Characterization of the hybrid Cell

**Figure 1:**
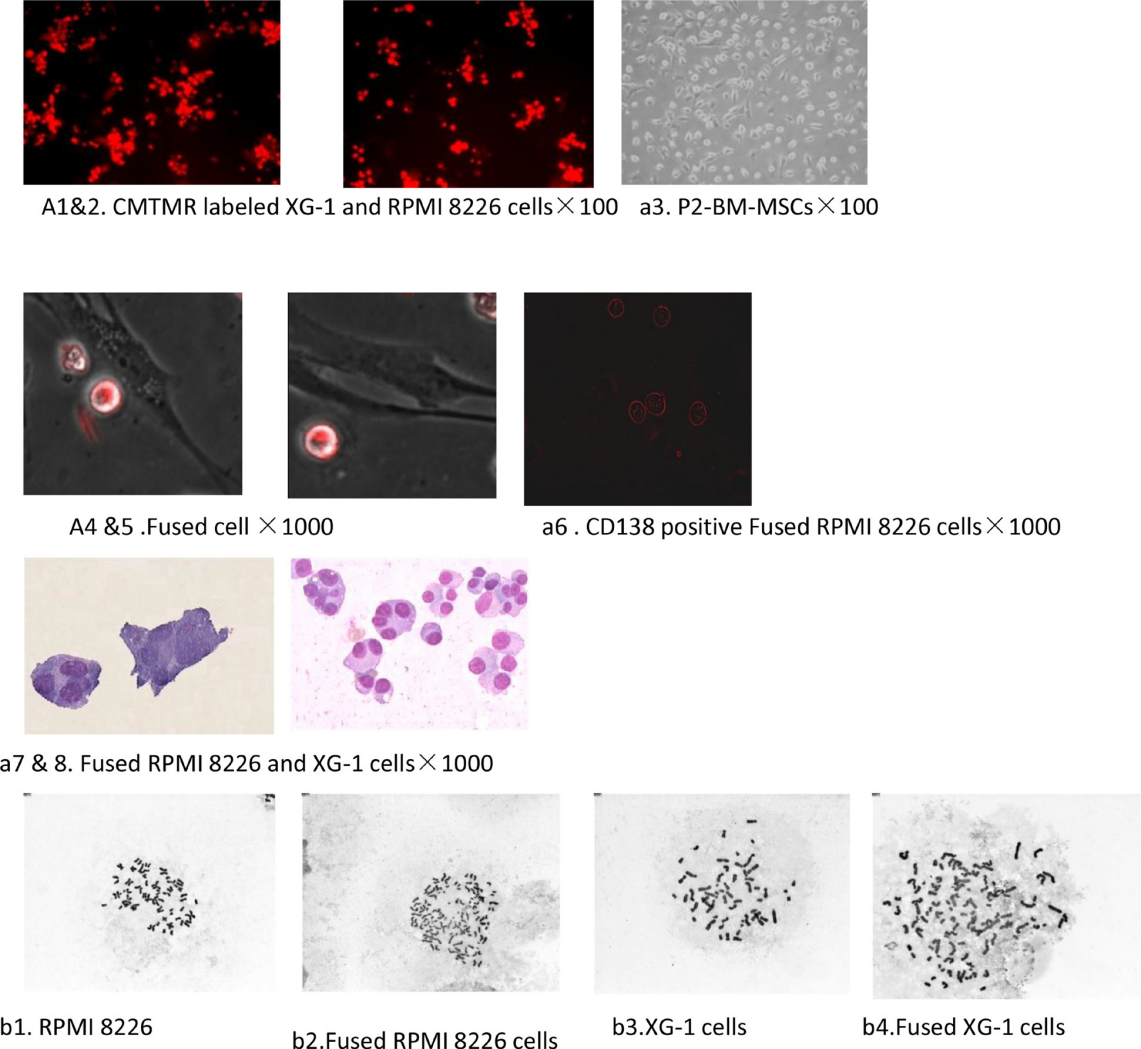
Cell fusion between hucMSCs and multiple myeloma cells (**a1**–**4**) The baseline characteristic of MM cells labeled with CMTMR fluorescent probes and BM-MSCs. (**a5**–**6**) The hybrid cell was detected on the second day after exposuring to PEG-1000. (**a7**): The fused cells were CD138 positve. (**a8**–**9**) The morphological characterization of the fused cell was observed under light microscope. The hybrid cells acquired larger size and multinucleation, in which partial chromatin condensation, a visible nucleolus, and one or more round or oval nucleus. (**b1**–**4**) Cytogenetic studies confirmed that there were numerical chromosome aberrations in fused cells than those in parental cells.

In the absence of specific biological or chemical induction signals, cells engaged in a physical contact do not normally fuse together. Employing an *in vitro* co-culture research model we showed that BM-MSCs and MM cells were fused in medium containing PEG-1000. Although the fusion efficiency of these two cells was very low in the experiments condition, the formation of polykaryons was confirmed under the light microscope. We got and isolated two clones of fusion cell from 23 experiments. Conversely, we did not get hybrid cells from the controls. A few cells isolated from controls was mainly MM cells and MSCs and these MM cells constantly adhere to MSCs *in vitro* (Figure 1a 1–6). Morphological observation showed that both MM cells and BM-MSCs lost their former morphologies. After fusion with BM-MSCs, the hybrid cells acquired larger size and multinucleation, in which partial chromatin condensation, a visible nucleolus, and one or more round or oval nucleus. There is a slight basophilic cytoplasm usually with neuritis and no granules. The fused cells were CD138 postive and did not exhibit a conspicuous spindle shape, which was different from the morphology of BM-MSCs and MM cells (Figure 1a 7–9). Cytogenetic studies confirmed that there were numerical chromosome aberrations in fused cells than those in parental cells (Figure 1b 1–4). The number of chromosome of PRMI8226 and XG1 before the fusion process was 47 ± 2.6 and 50 ± 3.2 and changed to 86 ± 12.6 and 91 ± 8.7 post-cell fusion, respectively. All this process might contribute to its genomic heterogeneity.

**Figure 2:**
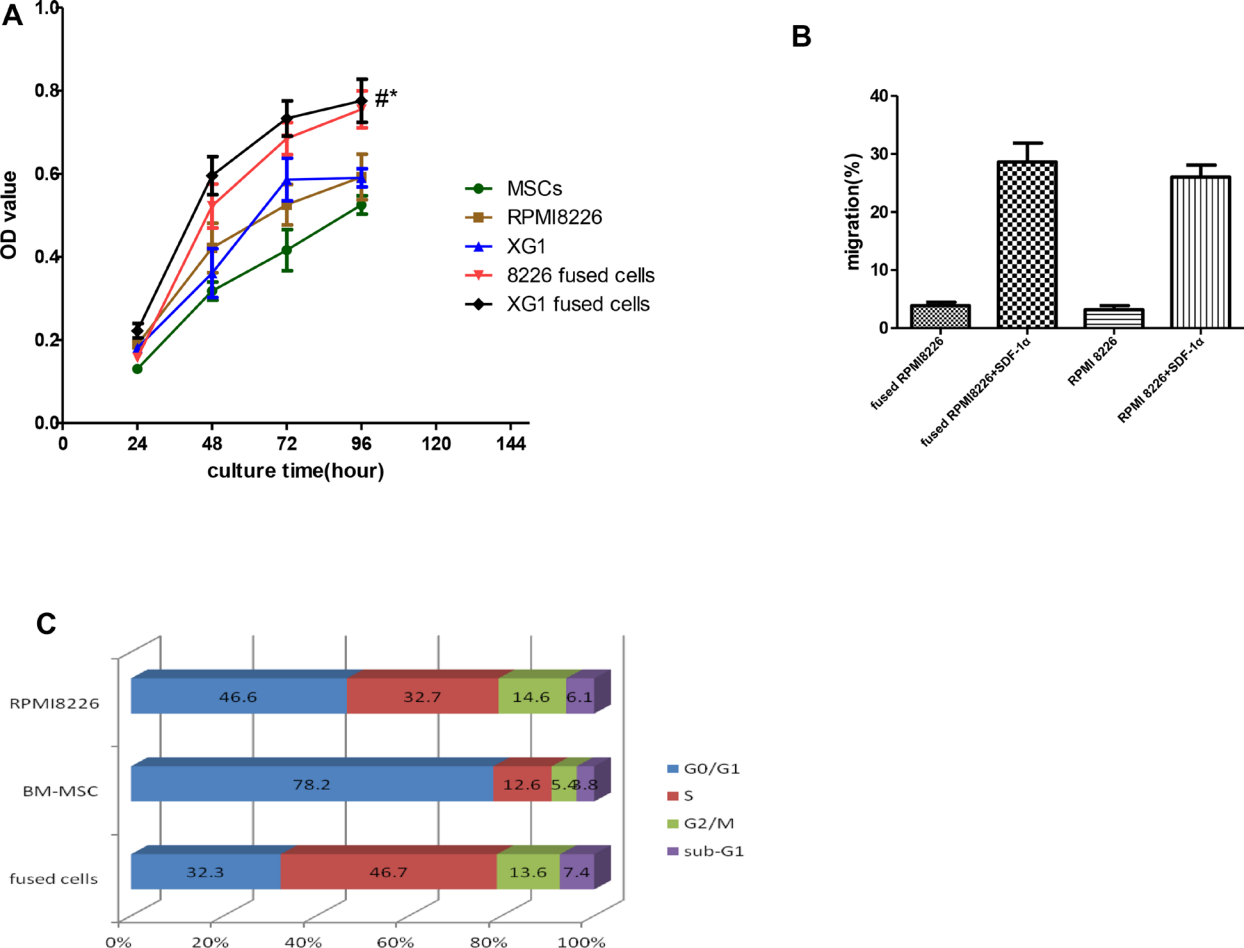
Effects of cell fusion on cell behaviors Fusion with BM-MSCs not only enhanced in growth and migration of the hybrid cells, but also promoted the cells into cell cycle *in vitro*. In the meantime, the cell fusion has the ability to promote more hybrid cells into cells cycle. (**A**) Effects of cells fusion on proliferation of hybrid cells. (^*^p < 0.05, compared to those with MM cells; and ^#^*p* < 0.01, compared to those with MSCs). (**B**) Effects of cell fusion on migration of hybrid cells in medium with or without SDF-1α (N: no statistic significance, *p* > 0.05). (**C**) Effects of cell fusion on cells cycle *in vitro*.

In order to further investigate the effect of cell fusion on cell growth ability, we compared *in vitro* growth rates of the hybrid cells with that of their parental MM cells by CCK-8 assay. At the fourth day after cell seeding, the number of hybrid cells was markedly higher than that of their parental cells (*p* < 0.05, Figure 2A). We also examined the migration ability *in vitro* by transwell migration assay in medium with or without SDF-1. Because of the morphological changes of MSC-MM cell hybrids, we hypothesized that the fused cells might be difficult to migrate through transwell membrance. In transwell migration assay, the number of both hybrid cells migrating through the transwell membrane was substantially higher compared to their cells, although there was no statistic significance (*p* > 0.05, Figure 2B). We also examined the changes of cell cycle of the hybrid cells by FCM and found that there were 32.3 ± 2.9% and 46.7 ± 2.5% fused cells in G0/G1 phase and S phase, respectively. In the meantime, BM-MSCs could have most of their cells in G0/G1 phase with fewer cells entering S phase. The percentages of BM-MSCs in G0/G1 phase and S phase were 78.2 ± 1.3% and 12.6 ± 0.9%, respectively. However, RPMI8226 cells in G0/ G1 phase and S phase remained at 46.6 ± 1.5% and 32.7 ± 2.4%, respectively. Our results showed that cell fusion has the ability to promote more hybrid cells into cells cycle (Figure 2C).

### Cell fusion increase the MM cells stemness

**Figure 3:**
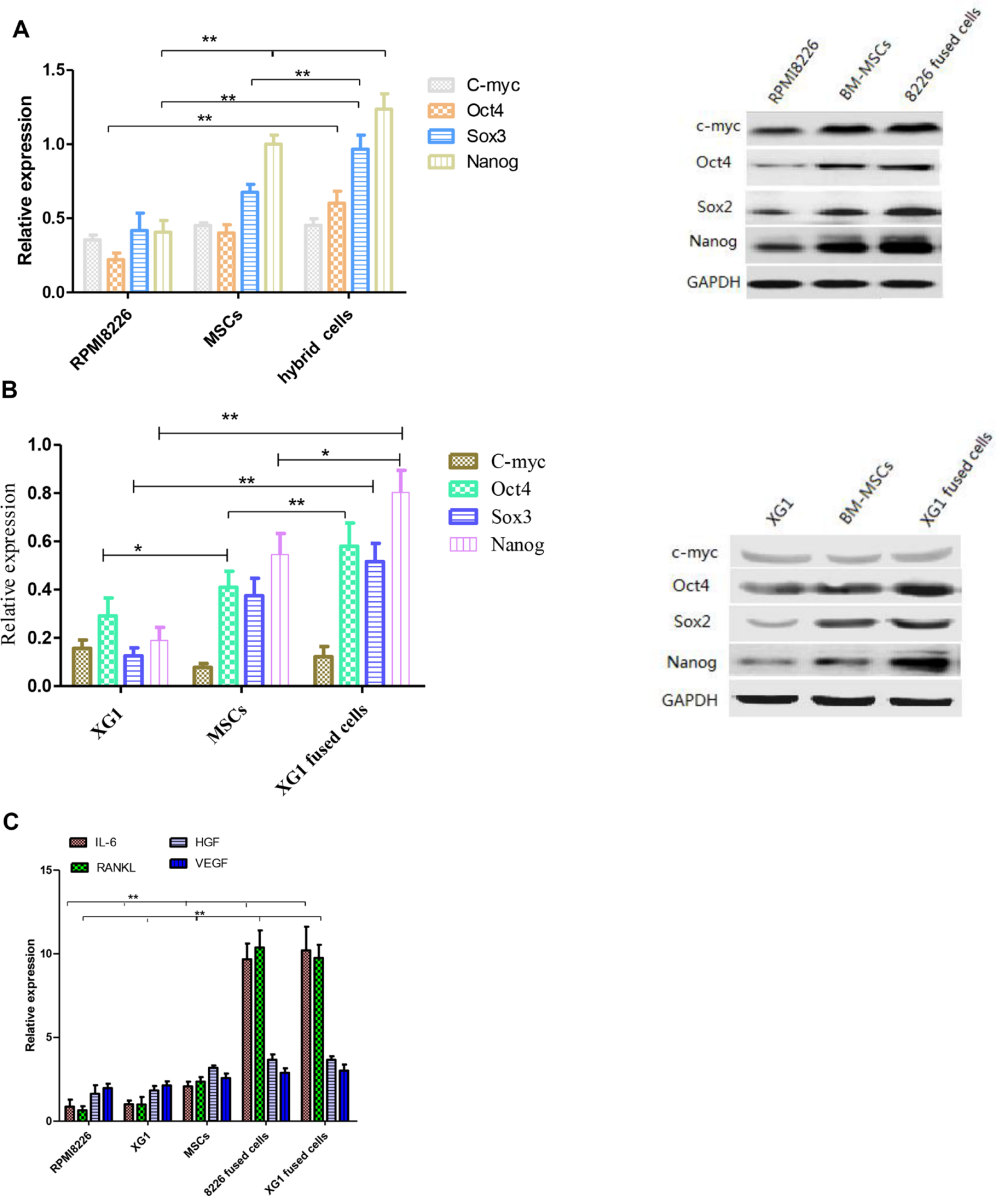
The expression of stemness genes and cytokines was analyzed before and after cell fusion (**A** and **B**) RPMI 8226, XG1 hybrid cells and parental cells reveal a differential expression of stemness-related marker proteins (^**^p < 0.01). (**C**) The expression of cytokines in RPMI 8226/XG1 hybrid cells related to the parental cells was determined by qPCR.

In order to investigate whether stem cells associated genes and cytokines genes are differentially expressed between the fused cells and the parental cells, both western blot and qPCR assays were used to determine the expression of those genes/cytokines in fused cells and parental cells. The expression of stemness factors including Oct4, Sox2, c-Myc and Nanog are known to be sufficient to reprogram somatic cells to pluripotent stem cells. Our results demonstrated that the expression of Oct4, Sox2 and Nanog were found to be significantly increased in fused cells compared to the parental MM cells by western blot (*p* < 0.01) (Figure 3A and 3B). It has been reported that MSCs secreted some cytokines, which played an important role in proliferation and survival of MM cells. Our results showed that the hybrid cells exhibited significantly increased expression of IL-6 and RANKL compared to their parental cells (*p* < 0.01), which might promote the fused cells to proliferate via a paracrine manner. There was no marked difference between the two fused cells we got. HGF and VEGF secreted by the fused cells were higher than those by their parental cells, but there was no statistic significance (Figure 3C). These results indicate that the hybrid cells may acquire multiple traits of stem cells.

### Fused cells exhibited increased drug resistance

**Figure 4:**
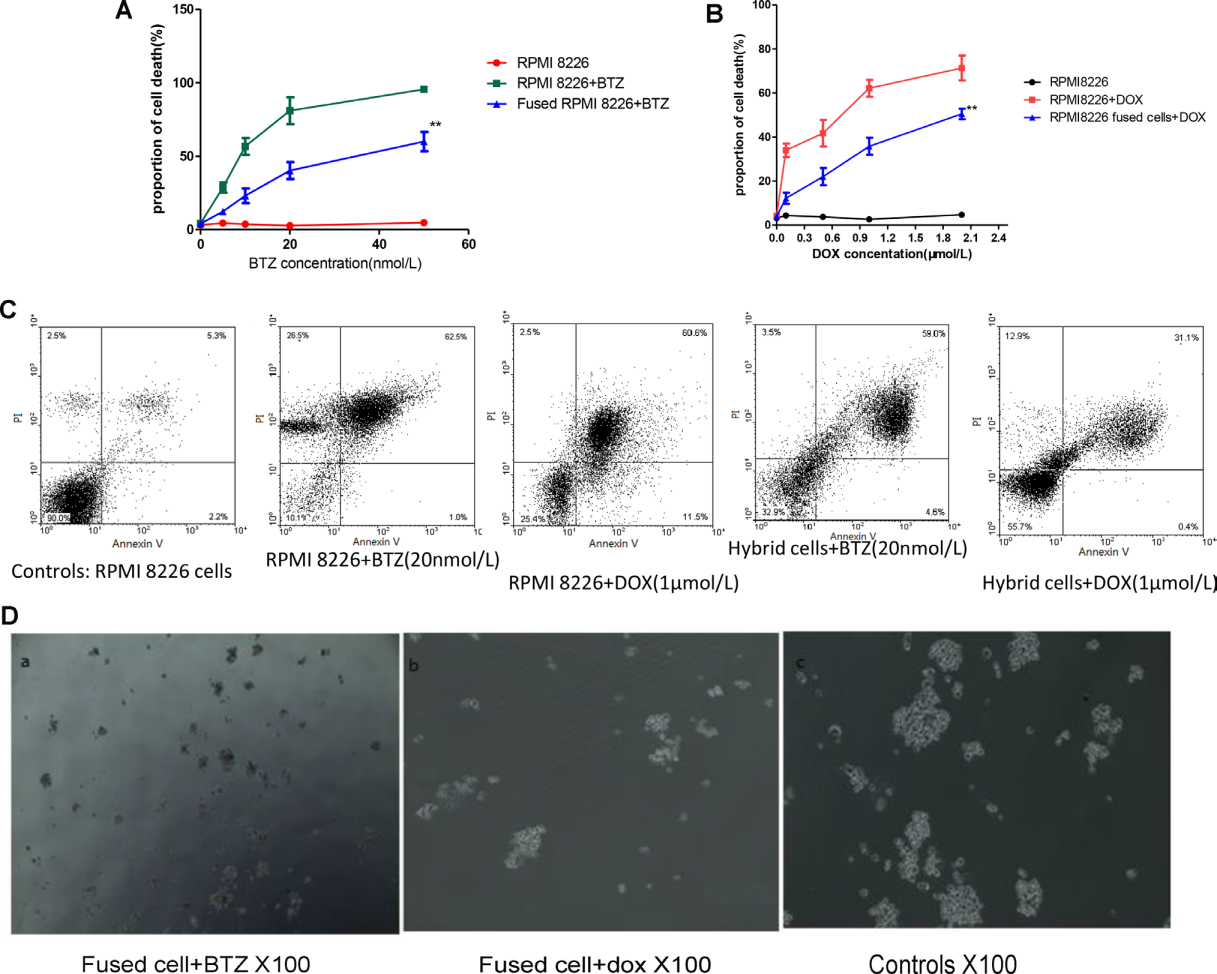
Cell fusion protected MM cells from apoptosis and promoted MM to chemoresistance (**A** and **B**) Drug resistant of MM cells and fused cell with different BTZ and DOX concentrations *in vitro*. The cell viability in fused cells groups was much more than those of their parental cells (*p* < 0.01). (**C**) Representative images of cell apoptosis in medium with BTZ or DOX were tested by flow cytometry. (**D**) The number of colony formed with hybrid cells in medium with BTZ or DOX were tested by clonogenic assay.

Drug resistance of the fused cells was also examined. The hybrid cells and RPMI 8226 cells were treated using different concentrations of DOX and BTZ for 24 h. Figure 4A illustrates that both hybrid cells and MM cells were sensitive to DOX or BTZ triggered apoptosis in dose depend manner. All the cells were more sensitive to the BTZ mediated apoptosis than that of DOZ. The survival rate of the fused cell was significantly higher than that of RPMI 8226 and the survival rates of the fused cell and RPMI 8226 decreased as DOX or BTZ concentration increased (Figure 4C). The percent of fused MM cell apoptosis was 56.2 ± 3.2% and 43.2 ± 2.7% in medium containing BTZ or DOX respectively, markedly lower than those of RPMI MM cells (*p* < 0.01), but higher than that of the controls (*p* < 0.01). In the present study, survival rate was defined by FCM and the results demonstrated that the fused cells exhibited the more of cell viability compared with their parental RPMI 8226 cells after DOX or BTZ treatments, which may contribute to MM chemoresistance (Figure 4A).

At the same time, clonogenic assay was also employed to evaluate the proliferation and drug resistance potential of the fused MM cells. These results showed that the RPMI 8226 themselves can form colony *in vitro* (Figure 4D). Interestingly, the number of colony formed with hybrid cells was very much augmented by this cell fusion process and was more than those with MM cells. The colonies formed in MM and fused cells were 28 ± 7 and 121 ± 11 respectively (*p* < 0.05). The number of colony decreased markedly to 8 ± 1.5 and 21 ± 9 when the cells were incubated in the medium containing BTZ (Figure 4B).

## DISCUSSION

Cell fusion occurs when cell membranes merge and the cytoplasm is mixed to form multinucleated cells. Cancer cells can fuse with normal cells (stromal, epithelial, and macrophages) and other cancer cells. The hybrid cells have novel properties and increase heterogeneity [21, 22]. It has been inferred that fusion events directly involving tumor cells may have great impact on metastatic behavior of certain types of cancer such as gastric cancer and breast cancer. This classic theory is called “cancer cell fusion” which first regard cell fusion event as a possible mechanism of tumor metastasis [23, 24]. By using FISH and immunohistochemistry on bone sections, Andersen TL and his colleagues [25] have found that the osteoclasts from myeloma patients contain nuclei with translocated chromosomes of myeloma B cells origin. Their results demonstrated that malignant cells can corrupt host cells via the cell fusion and/or the transfer of malignant DNA to enable the hybrid cells with novel characteristics. In this report, we fused human BM-MSCs with MM cell lines RPMI8226 and XG1 by PEG1000 to obtain hybrids *in vitro*. PEG is a widely used agent for cell fusion because of its simplicity and low cost. Moreover, cell fusion mediated by PEG is an efficient procedure for obtaining somatic cell hybrids and widely used in monclonal antibody production. Our results on artificially fusion of BM-MSCs and MM cells demonstrated a possible mechanism of the tumor-initiating cell generation in the human body. The fusion of BM-MSCs and MM cells enables the hybrids to exhibit stemness and MM characteristics. The tumorigenic hybrids express higher level of Oct4, Sox2, and Nanog compared to the parental MM cells by western blot and qRT-PCR and exhibited an enhanced invasiveness and motility in transwell assay. In the meantime, the cytokine profiles changed in fused cells. This indicates that the tumorigenic hybrids may get some novel characteristics through cell fusion event.

Recent works reveal that the presence of subclones of malignant plasma cells contribute to disease relapse, refractory and progression [26], which puts forward the cell fusion hypothesis of cancer stem cells. Clonal envolution is an essential step in the process of MM cells drug resistance and survival. The comparison of multiple myeloma cases at diagnosis and after treatment, Rashid NU and his colleagues’ study supports the concept of branching clonal development in a subset of patients [27]. Other proposed models of clonal evolution include no change, subclonal shift and linear evolution [28–30]. Our results showed that the fused cells in similar as for other cancer have complex genetic abnormalities including both structural and/numerical chromosome aberration. Although none of these chromosomal abnormalities is predictive of disease progression, the hybrid cells exhibited a marked proliferation and colony formation ability *in vitro*. Therefore, if chromosomal instability is currently regarded as a hallmark of MM, cell fusion may be claimed as a possible causative mechanism. Therefore, the artificial generation of fused tumor cells can be used to produce stem cell-like malignant cells for drug screening and studies on its mechanism.

In summary, we demonstrated that fusion of bone marrow mesenchymal stem cells and MM cells could produce to a subpopulation of hybrid cells showing an altered phenotype, including the morphological changes, numberical chromosome abnormality, up-regulated stem cell related genes expression as well as the enhanced ability to proliferaiton and migration. These properties could contribute it chromosomal instability and play a role on disease progression

## MATERIALS AND METHODS

### Culture of MM cell lines

The human multiple myeloma cell line RPMI 8226 was obtained from American Type Culture Collection (ATCC, Manassas, VA, USA). The cells were grown in RPMI 1640 medium supplemented with 10% fetal calf serum (FCS, Hyclone, Loan, UT, USA). The human MM cell line XG1 was a kindly gifts of Professor Xueguang Zhang (Soochow University, Suzhou) and was cultured in RPMI 1640 medium supplemented with 10% FBS and 3 ng/mL interleukin-6 (IL-6) (Cytolab, Lynnwood, WA, USA).

### Isolation, expansion and characterization of BM-MSC

Isolation, expansion and characterization of normal bone marrow(BM) MSCs was obtained with a given informed consent and approved by the hospital ethics board. BM aspirates were obtained from healthy donors with a median age of 34 years by puncturing the iliac crest. The mononuclear cell (MNC) fraction was isolated by Ficoll (GE Healthcare, Walkersville, MD 21793-0127, USA) density gradient centrifugation at 420 g for 30 min at room temperature (RT). MNCs in the interphase were washed twice with phosphate-buffered saline (PBS) and seeded in DMEM-LG+10% FCS on FCS-precoated, six-well plates with 3 × 10^6^ MNC/cm^2^. The BM-MSCs were passaged at 70% confluence with trypsin/EDTA (1:250, PAA), by seeding 200 cells/cm^2^ for BM-MSCs.

### Cytogenetic analysis

Karyotyping of MM cells was performed by a standard method described elsewhere [31]. Briefly, Parental cells and hybrid cells (5 × 10^6^) were cultured for 4 h with 0.2 μg/ml Colcemid solution (Sigma, USA) in a humidified atmosphere at 37° C and 5% CO_2_. The cells were harvested, washed once with PBS and were resuspended in 75 mM KCl for 30 min. Subsequently, the resulting cells were fixed in methanol and acetic acid (3:1) and were carefully washed twice with same solution. Pipette three drops of the cell suspension onto a clean and wet slide and dry at RT. Finally, the number of chromosomes were checked under microscopy.

### Cell labeling

In order to monitoring the fusion process, the MM cell lines were incubated with orange (CMTMR, 5-(and-6)- (((4-Chloro- methyl)Benzoyl) Amino)Tetramethylrhodamine) fluorescent probes according to the manufacturer’s instructions (Invitrogen, Carlsbad, CA, USA). This dye is fluorescent chloromethyl derivatives that freely diffuse through the membranes of live cells, but once inside the cell it is converted into membrane-impermeant reaction products, facilitating the identification of cell fusion products. Stained cells were continuously observed in phase contrast/fluorescent microscopy to monitor cell fusion. To confirmed the fusion process occurred between the MM cells and BM-MSCs, the fused cell stained with CD138 and checked under fluorescence microscope.

### Cell culture and induction cell fusion

The second passaged BM-MSCs were routinely cultured in DMEM-LG (Gibco BRL, Grand Island, NY, USA) and supplemented with 10% heat-inactivated FCS. When adherent cells reached 80% confluence, cells were isolated by treatment with 0.25% trypsin/ethylenediaminetetraacetic acid (EDTA) and washed twice with PBS. Total cell counts as well as the respective proportion of viable and dead cells were enumerated by Trypan blue dye exclusion using a phase-contrast microscope. The resulting cells were mixed with CMTMR labeled MM cells at ratio of 2 to 1, then the pre-heated PEG-1000 was added to the mixture at a final concentration of 50% (v/v) for an exposure time of either 1, 3 and 5 minutes in a water bath at 37° C. Because the MM cells tend to fuse spontaneously, We processed and cultured the mixed cells at same condition without PEG-1000 as a negative control. During this time, the tube was gently swirled to keep cells in suspension. L-DMED containing 10% FCS was used to stop the interaction and cells were then collected, extracted with centrifugal and washed twice with PBS, then the cells were resuspended at a concentration of 10^6^ cells/mL in DMEM-LG with 10% FCS. The cells were plated 6-well at a concentration of 10^6^/ml. The cultures were fed every 72 hours with the fresh media. After each PEG treatment, cell fusion products were evaluated and counted by phase contrast/fluorescence microscopy. Cell viability was determined by trypan blue exclusion.

### Isolation of fusion cells

We used a sedimentation chamber previously described by Pedrazzoli *et al.* [32] for the isolation of cell fusion products. Briefly, the separation chamber was filled from the bottom with a linear gradient (1%–3%) of human albumin in RPMI1640, generated with a gradient mixer and peristaltic pump. Then, the cell sample was seeded onto the gradient by reversing the peristaltic pump, thus lowering the cell sample to the cylindrical part of the device. Cells were allowed to sediment at unity gravity for 3 h, and then isolated fractions of 15 ml each were collected from the bottom of the sedimentation chamber. The percentages of fusion cells in each fraction were evaluated by fluorescence microscopy.

### Culture and proliferation of isolated cell fusion products

The resulting fused cells were maintained at 37° C in DMEM-LG with 10% FCS for 48 h. The cells were passaged at 80% confluence by seeding 400 cells/cm^2^ for fused cells. Morphological examination and chromosomal analysis were done to confirm the cell fusion.

### Cell proliferation and migration assays

Fused RPMI8226 cells and RPMI8226 cells (1 × 10^4^/100 μ L/well) were seeded into 96-well culture plates (Corning, USA) at 37° C for 24, 48, 72 and 96 h. The cells were incubated with CCK-8 (10 μL/well) (Dojindo, Japan) for 3 h. Then, optical density (OD) values were determined by an enzyme-labeled instrument.

We performed a transwell migration assay (Costar, Cambridge, MA, USA) using the fusion cell in the presence of BM-MSCs, which were cultured in the lower chambers. Three groups were included in the assay: (1) control, upper chamber: fused RPMI8226 cells, and lower chamber: DMEM-LG supplemented with 0.5% FBS; (2) MM cell, upper chamber: RPMI8226 cells, and lower chamber: DMEM-LG supplemented with 0.5% FBS and 50 ng/ml SDF-1α (Peprotech, Rocky Hill, NJ, USA); (3) fusion cell, upper chamber: fused RPMI8226, and lower chamber: DMEM-LG supplemented with 0.5% FBS and 50 ng/ml SDF-1α. In brief, the fused cells and RPMI8226 cells were suspended in 0.5% FBS medium, and 5 × 10^5^ cells were placed in the upper chambers of the transwell plates with or without SDF-1**α**in the lower chambers. After 4 h at 37° C, cells that migrated to the lower chambers were counted. Triplicate experiments were performed in each group, and the means and standard deviations were calculated.

### Cell cycle analyses

The cell cycle status was detected by flow cytometry(FCM) using a protocol described previously [33] and analyzed by CellQuest software (BD Biosciences Pharmingen, San Jose, CA, USA). Briefly, one million of harvested fusion cells were fixed with 70% cold ethano at 4° C for 30 min, washed with PBS twice, followed by treatment with 400 μg/mL RNAse A for 20 min at 37° C, and stained with 3 μg/mL PI (Sigma, St. Louis, MO, USA) at RT for 30 min. DNA content was analyzed by FCM.

### Drug resistance and colony assay

The fused cells and RPMI 8226 cells were first incubated in a 6-well plate for 15 h. After this step, different concentrations of doxorubicin (DOX, Jinyao company, Tianjin, China) or bortezomib (BTZ, BSP Pharmaceuticals SRL, Latina, Scalo, Italy) was added to the culture medium with three parallel samples for every group. The cultures were continued for 48 h and cell numbers, viability (trypan blue assay) and annexin V/PI binding were determined by FCM.

A series of colony assays were performed with fused cells and RPMI 8226 cells. The cells were seeded directly into-24 well plates and overlaid with 1% methylcellulose gel with 10% FCS in the presence or absence of BTZ. The number of colonies in each well was counted after 2 weeks under microscopic examination, with a colony defined as a cluster of at least 50 cells. The results are the mean values of three independent experiments. The number of colonies in each well was counted after 2 weeks under microscopic examination, with a colony defined as a cluster of at least 50 cells.

The concentrations of DOX and BTZ used in this study were 0.1, 0.5, 1 and 2 μmol/L and 5, 10, 20 and 50 nmol/L respectively.

### Real-time quantitative PCR

The changes of key transcription factors(Oct4, c-Myc, Sox2 and Nanog) and growth factor(IL-6, RANKL, HGF and IGF-1) gene expression of the fused cells and parental cells were also detected with western blot and real-time quantitative PCR (qPCR) assay. Briefly, total mRNA was isolated from the fusion cells by using Trizol reagent according to the protocol offered by the manufacturer. The expression of these genes was determined by qPCR using the SYBR Green Master Mix Kit. The sequences of the primers were listed in Table 1. Thermocycler conditions included an initial hold at 50° C for 2 minutes and then 95° C for 10 minutes, which was followed by a two-step PCR program of 95° C for 15 seconds and 60° C for 60 seconds repeated for 40 cycles by using a Mx3000P QPCR System (Stratagene, USA). GAPDH primers were used to normalize the samples. Data were analysed using the 2^−(ΔCt)^ method. The freshly isolated MM cells and RPMI 8226 MM cells were used as controls.

**Table 1:** Primer sequences for qPCR

gene	Forward primer (5′–3′)	Reverse primer (5′–3′)
GAPDH	GCA CCG TCA AGG CTG AGA AC	TGG TGA AGA CGC CAG TGG A
c-Myc	CCC GCT TCT CTG AAA GGC TCT C	CTC TGC TGC TGC TGC TGG TAG
Nanog	CAG AAG GCC TCA CAC CTA C	ATT GTT CCA GGT CTG GTT GC
Sox2	ACA CCA ATC CCA TCC ACA CT	GCA AAC TTC CTG CAA AGC TC
Oct4	CAC TGT ACT CCT CGG TCC CTT TC	CAG GCA CCT CAG TTT GAA TGC
IL-6	TTC CTC ACC ACT GAA TCT ACA GAA	CTT TGG AGG AGT GTG AGG TG
RANKL	AGC ACA TCA GAG CAG AGA AAG C	CAG TAA GGA GGG GTT GGA GAC C
HGF	TTG GTG GAC GAT GAC ACG TG	GTG TCT CCC AAC ATG TCC ATG
VEGF	CGG CTT GTC ACA TTT TCT GG	CAA GGC TCA CAG TGA TTT TCT GG

### Immunoblotting

Cells were lysed in RIPA buffer supplemented with proteinase inhibitor. The proteins were extracted and bicinchoninic acid was used to determine protein concentrations. The lysates were separated by sodium dodecyl sulfate-polyacrylamide gel electrophoresis (SDS-PAGE; Bio-Rad, Hercules, CA, USA), and transferred to polyvinylidene difl uoride membranes, which were subsequently blocked in Tris-buffered saline containing 5% non-fat milk and 0.1% Tween for 1 h. The membranes were then incubated with polyclonal rabbit anti-Oct4, anti-Sox2, anti-Nanog, anti-c-Myc(Cell Signaling Technology, Danvers, MA, USA) and anti-glyceraldehyde 3-phosphate dehydrogenase (GAPDH) (Sigma) at 4° C overnight. Antigen-antibody complexes were detected using secondary antibodies conjugated to horseradish peroxidase (Invitrogen, Carlsbad, CA, USA) and visualized by enhanced chemiluminescence (GE Healthcare, Little Chalfont, UK)

### Ethical approval

Our study programs were approved by the Ethics Committee of Soochow University Hospital. According to Soochow University Hospital committee guidelines, formal written consent was obtained from all participants prior to performing the studies.

### Statistical analyses

Unless indicated otherwise, all values are expressed as mean ± SE. Statistical analysis was performed using GraphPad Prism 5 software (San Diego, CA, USA). Statistical significance was determined using the nonparametric Mann–Whitney *U* test or Student’s t test. A *p*-value of < 0.05 was considered significant. All experiments were conducted at least 3 separate times.
